# Examining Students’ Acceptance and Use of ChatGPT in Saudi Arabian Higher Education

**DOI:** 10.3390/ejihpe14030047

**Published:** 2024-03-17

**Authors:** Abu Elnasr E. Sobaih, Ibrahim A. Elshaer, Ahmed M. Hasanein

**Affiliations:** 1 Management Department, College of Business Administration, King Faisal University, Al-Hassa 31982, Saudi Arabia; ielshaer@kfu.edu.sa (I.A.E.); aabdelrazek@kfu.edu.sa (A.M.H.); 2 Faculty of Tourism and Hotel Management, Helwan University, Cairo 12612, Egypt; 3 Faculty of Tourism and Hotel Management, Suez Canal University, Ismailia 41522, Egypt

**Keywords:** ChatGPT, Chatbot, artificial intelligence, technology acceptance, technology use, Saudi Arabian education

## Abstract

This study examines students’ acceptance and use of ChatGPT in Saudi Arabian (SA) higher education, where there is growing interest in the use of this tool since its inauguration in 2022. Quantitative research data, through a self-reporting survey drawing on the “Unified Theory of Acceptance and Use of Technology” (UTAUT2), were collected from 520 students in one of the public universities in SA at the start of the first semester of the study year 2023–2024. The findings of structural equation modeling partially supported the UTAUT and previous research in relation to the significant direct effect of performance expectancy (PE), social influence (SI), and effort expectancy (EE) on behavioral intention (BI) on the use of ChatGPT and the significant direct effect of PE, SI, and BI on actual use of ChatGPT. Nonetheless, the results did not support earlier research in relation to the direct relationship between facilitating conditions (FCs) and both BI and actual use of ChatGPT, which was found to be negative in the first relationship and insignificant in the second one. These findings were because of the absence of resources, support, and aid from external sources in relation to the use of ChatGPT. The results showed partial mediation of BI in the link between PE, SI, and FC and actual use of ChatGPT in education and a full mediation in the link of BI between EE and actual use of ChatGPT in education. The findings provide numerous implications for scholars and higher education institutions in SA, which are also of interest to other institutions in similar contexts.

## 1. Introduction

Since the inauguration of Chat Generative Pre-trained Transformer (ChatGPT) in 2022, there has been growing interest among students in using ChatGPT in education [1]. ChatGPT is a chatbot generated by the OpenAI, which assists users in creating human-like text by processing commands and conversations [2]. Studies on the use of ChatGPT in the context of higher education, see for example, refs. [1,2,3,4], have shown several benefits to using ChatGPT in education, such as receiving quick and prompt service, and academic and nonacademic support; developing language skills; and using editing services. Previous research has also shown that ChatGPT assists students in their assignments, projects, homework, and research tasks. However, there are some concerns among scholars and higher education leadership about the incorporation of ChatGPT in higher education and its use for supporting teaching and learning [5,6,7,8]. Other concerns include generation of biased information, fake citations, and reliability and intellectual property concerns, which raise ethical alarms about content provided by students. Hasanein and Sobaih [1] commented on the destructive influence of ChatGPT on academic integrity and the misuse of information provided by ChatGPT. Furthermore, there are other concerns among many scholars, e.g., refs. [1,9], about the integration of ChatGPT into education; said concerns relate to learning outcomes and students’ skills, particularly critical thinking, which are expected to be negatively affected. Therefore, it has been argued that caution should be taken by academic institutions to prevent over-dependence by students on using ChatGPT for academic purposes [1].

There have been some attempts by scholars, e.g., refs. [2,6], to understand the adoption of AI, e.g., ChatGPT, in education. Nonetheless, there have been few attempts (despite growing numbers of studies) to understand the variables that drive students’ use of ChatGPT in education [1,4,10]. The study of Alotaibi and Alshehri [10] delved into the potential advantages and obstacles associated with integrating AI-driven learning outcomes within Saudi Arabia’s higher education system. Their findings underscored that AI is still in an early developmental phase in the realm of education, yet its significance for higher education institutions is undeniable. Embracing this transformative technology is essential for addressing forthcoming learning challenges. Equally crucial is ensuring that all Saudi Arabian students develop the requisite technical competencies to engage with and contribute to the field of artificial intelligence in the future.

Strzelecki [4] identified a research gap regarding students’ acceptance and use of ChatGPT in education. Hasanein and Sobaih [1] conducted in-depth interviews to explore key stakeholders’ perceptions, including students, about the drivers of using ChatGPT in higher education, and they identified 12 factors affecting students’ usage of ChatGPT for academic-related reasons. These factors are ease of use, homework assistance, quick response, language editing, problem solving, exam preparation, data analysis, research support, adaptive learning, defining concepts, learning resources, and assessment activities. Strzelecki [4] adopted “Unified Theory of Acceptance and Use of Technology (UTAUT2)” to examine the variables that influence higher education students’ acceptance and use of ChatGPT in Poland, albeit calling for future research studies in other contexts from the students’ perspective. This study attempts to fill this research gap in the Saudi higher education context and explore the factors that affect students’ use of ChatGPT for learning.

This research examines students’ acceptance and use of ChatGPT in Saudi Arabian higher education institutions, where students have become interested in the use of this tool for academic purposes. Understanding the variables that affect students’ acceptance and use of ChatGPT in education will enable leaders of universities and scholars to gain better understanding of these factors and carry out superior management of students’ incorporation of AI tools for academic purposes. This research adopted the UTAUT framework for better understanding these factors among Saudi students in higher education. For this reason, the next part of the article reviews the UTAUT framework and discusses the theoretical foundation of the study. It then explains the methods adopted in the study and presents and discusses the results. Final remarks are then presented and guidelines for further studies are suggested.

## 2. Literature Review

### 2.1. Students’ Acceptance of ChatGPT and Behavioral Intentions

UTAUT serves as a comprehensive background for investigating the acceptance and utilization of technological innovations in various contexts [11,12,13]. Applying the UTAUT framework to integrate ChatGPT in educational settings provides a structured approach to understanding students’ behavioral intentions (BIs) regarding this advanced language model [14,15]. According to Venkatesh et al. [13], the key determinants influencing users’ accepting and using behaviors encompass performance expectancy (PE), effort expectancy (EE), social influence (SI), and facilitating conditions (FCs). PE is related to individuals’ perceptions of the model’s capabilities in enhancing their learning experience [13]. Students’ perceptions of ChatGPT’s capabilities and its potential to contribute positively to their learning experiences directly influence their BIs [15,16,17,18,19,20]. Furthermore, Al-Emran et al. [21] revealed that when students believe that ChatGPT can effectively assist them in learning complex topics, developing creative ideas, or boosting overall academic achievement, they are more likely to show a positive BI to utilize ChatGPT for various educational purposes. With regard to EE, it refers to users’ perspectives concerning the degree of simplicity or complexity of using the technology [13,22]. It plays a key part in shaping people’s willingness and intention to engage with the language model [12]. Menon and Shilpa [15] argued that students’ perception of ChatGPT as user-friendly, easily understandable, and seamlessly integrated into daily activities positively influences their BIs. Moreover, recent studies, e.g., refs. [4,21,23] have revealed that students’ perception has been influenced by several elements such as the model’s ease of interaction, user interface design, and overall user experience. When students anticipate minimal effort in using ChatGPT, this increases their likelihood of integrating ChatGPT into their daily educational routine [1,24,25].

SI considers the impact of peers and instructors in shaping students’ attitudes towards ChatGPT [13,22]. It manifests when students observe the attitudes, opinions, and behaviors of those around them in relation to the incorporation of ChatGPT into education [12,14]. Several studies, e.g., refs. [15,26,27], have argued that colleagues who spread positive information about utilizing ChatGPT for educational activities are likely to positively affect the BI of other students. Regarding FCs, this factor assesses the availability of necessary resources for its effective use [13]. It encompasses elements like the accessibility of the technology, the provision of necessary tools and training, and the overall institutional support for its integration [4,15,28]. This should have a significant influence on students’ BIs to use ChatGPT when they recognize that the educational setting offers the necessary resources and support for its effective utilization [1,29]. The study of Al-Emran et al. [21] argued that FCs go beyond simply necessary technological resources; they entail the creation of a supportive infrastructure that enables users to navigate and harness ChatGPT effectively, thereby bolstering students’ BIs to engage with the technology. Therefore, based on these discussions, we hypothesize that

**H1:**  *PE has a significant positive effect on BI to use ChatGPT in education*.

**H2:**  *EE has a significant positive effect on BI to use ChatGPT in education*.

**H3:**  *SI has a significant positive effect on BI to use ChatGPT in education*.

**H4:**  *FC has a significant positive effect on BI to use ChatGPT in education*.

### 2.2. Students’ Acceptance and Actual Use of ChatGPT

Several studies, e.g., refs. [15,21,23,24], on chatbots (e.g., ChatGPT) have consistently highlighted the pivotal role of PE and EE in predicting student acceptance and actual use of such innovative technology. These crucial determinants are affected by various variables related to the chatbot’s perceived usefulness, ease of use, simplicity, and compatibility with user needs [17,18]. Additionally, several aspects such as task achievement, sense of achievement, and heightened engagement have been identified as key contributors to PE [19]. In terms of educational context, Menon & Shilpa [15] argued that PE is considered a predominant factor affecting students’ perceptions of using ChatGPT for educational purposes. Moreover, recent research [1] has revealed that understanding EE toward ChatGPT, i.e., user-friendliness, simplicity, adoption, and integration into daily routines, is more likely, leading to increased actual usage and the greater value of ChatGPT in educational contexts. Regarding SI toward ChatGPT in educational contexts, recent studies, e.g., refs. [1,14,21,23,26], have observed that positive attitudes, endorsements, or active usage of chatbot-based AI within social networks (e.g., colleagues) are likely to positively influence people’s actual use of ChatGPT. Additionally, recommendations, encouragement, or shared positive experiences from others have an important impact on users’ decision to actively incorporate ChatGPT into their tasks and routines [1,15,21]. Previous research, i.e., refs. [15,21,25,30,31], that has measured the adoptability of ChatGPT in education has underscored the substantial impact of FCs on its adoption and real usage. Furthermore, the research conducted by Menon and Shilpa [15] has shown that FCs, i.e., a mobile device, consistent internet connection, and technical support, are pivotal factors that can profoundly affect the adoption of ChatGPT. Hence, we could postulate that

**H5:**  *PE has a significant positive effect on Use of ChatGPT*.

**H6:**  *EE has a significant positive effect on Use of ChatGPT*.

**H7:**  *SI has a significant positive effect on Use of ChatGPT*.

**H8:**  *FCs have a significant positive effect on Use of ChatGPT*.

### 2.3. BI and Use of ChatGPT

The association between BI to use ChatGPT and actual usage is a critical aspect of the technology’s adoption and utilization. Venkatesh et al. [13] defined BI as the willingness and readiness of individuals to engage with a particular technology. This intention is often considered a direct precursor to use of the technology [22,32]. Numerous studies, i.e., refs. [15,21,23,31,33,34] showed that when students convey a positive BI towards ChatGPT, signifying their readiness to include it in their routines or tasks, there is a heightened probability this intention being translated into actual use. Furthermore, the more substantial the intention to use ChatGPT, the more likely students are to engage in active interaction with the platform, seek its assistance, and integrate it into their academic activities [13,29]. Therefore, based on these discussions, we are prompted to hypothesize the following:

**H9:**  *BI positively influences use of ChatGPT*.

### 2.4. The Role of BI in the Link between Students’ Acceptance and Usage of ChatGPT

Undoubtedly, students’ BI to use ChatGPT has a pivotal role in the relationship between their acceptance of using this innovative technology and its actual usage in higher education [15,21,35]. However, it is noteworthy that there is a research gap concerning the mediating impact of BI on the link between students’ acceptance and usage of ChatGPT in higher education. This research attempts to address this gap in research and draws on the UTAUT to hypothesize that

**H10:**  *BIs mediate the link between PE and Use of ChatGPT*.

**H11:**  *BIs mediate the link between EE and Use of ChatGPT*.

**H12:**  *BIs mediate the link between SI and Use of ChatGPT*.

**H13:**  *BIs mediate the link between FC and Use of ChatGPT*.

**Figure 1:**
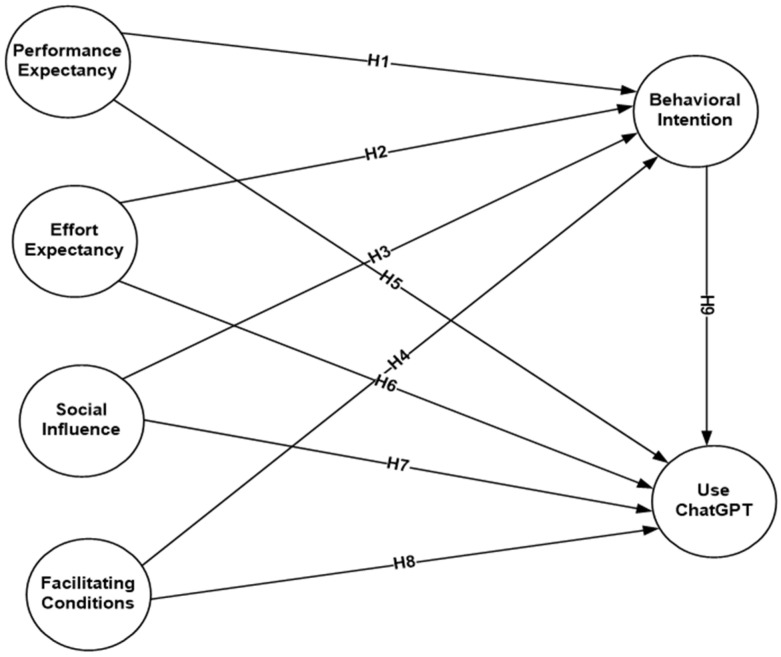
The conceptual model.

A summary of all the relationships in the research conceptual framework is presented below (Figure 1).

## 3. Methods

### 3.1. Measures and Scale Development

The survey adopted in this research has three sections. The first section outlines the research objectives and guidelines needed for completing the survey. Throughout the second section, information about participants’ profile is gathered. The third section of the study involved investigating various aspects, with a rating of 1 representing “strongly disagree” and a rating of 5 indicating “strongly agree”. The ChatGPT UTAUT scale was adopted from Venkatesh et al. [13] and includes four variables: PE, EE, SI, and FC. In addition to these variables, the ChatGPT TPB scale also includes a behavioral intention variable adopted from Ajzen and Fishbein [36] as well as an intent-to-use variable developed from previous studies [22]. To verify the consistency and ease of use of the survey, the survey was subjected to evaluation by professors and education leaders. In order to maintain the validity of the survey’s content, certain statements’ wording was modified and arranged based upon the responses of participants and scholars [37].

### 3.2. Research Sample and Data Collection Method

The current research focused on students who utilize ChatGPT in their educational activities within public universities in Saudi Arabia. To gather the necessary data, a convenience sampling method was used, specifically targeting students from the School of Business at King Faisal University. The gathering of responses commenced at the start of first semester for study year 2023–2024, which corresponds to September 2023, and continued for a duration of one month. Among 600 surveys distributed, a total of 520 were successfully completed, resulting in a remarkable response rate of 87%, and notably, there were no missing data. The sample size of 520 valid responses adhered to Nunnally’s criteria [36] for maintaining a 1:10 item-to-sample ratio. In this context, since there were 21 items in the survey, a sample size of at least 210 participants was considered appropriate. Among the 520 valid responses, it was observed that 54.8% of the students identified as male, amounting to a total of 285 students, while 45.2% of the students identified as female, totaling 235 students. The age group that constituted the majority of students, accounting for 51.3% of the respondents, was from 20 to 25. Within this age group, 54.1% of the students were classified as junior and senior students.

### 3.3. Data Analysis

The data were subjected to analysis utilizing PLS-SEM, a variance-based algorithm employed for analyzing paths. PLS-SEM offers an unconventional alternative to the classical covariance-based SEM (CB-SEM) [38]. Acknowledged for its applicability in prediction-focused and exploratory research, PLS-SEM has risen in prominence [39]. It operates without the constraint of normality assumptions in the sample spreading and has demonstrated efficacy with both small and large sample sizes [38,40]. The preference for exploratory research and the adaptable nature of the method in handling diverse sample sizes were the driving factors behind choosing this approach for our study. The PLS analysis was executed utilizing SmartPLS 4 [41]. Model estimation involved a bootstrapping technique with 5000 resamples using Mode A (reflective mode) [40]. Furthermore, to mitigate common-method variance (CMV), following Podsakoff et al. [42], an analysis was performed using Harman’s one-factor test. All 25 items were subjected to an exploratory factor analysis (EFA), demonstrating that the first variable clarified only 35.28% of the total variance. This suggests that CMV was not a prevalent concern in the present study. Additionally, all variance inflation factor (VIF) scores were below 0.5, indicating the absence of multicollinearity issues (Table 1).

## 4. Results of the Study

The assessment of the outer model (measurement model) included calculation of the psychometric properties of diverse scales using criteria such as Cronbach’s α, composite reliabilities (CRs), and average variance extracted (AVE). All scale items demonstrated standardized loadings of 0.7 and higher, signifying robust convergent validity. Furthermore, all values of Cronbach’s α and CRs exceeded the minimum threshold of 0.7, indicating the internal consistency of items and constructs (Table 2). The AVE results for all constructs surpassed the recommended threshold of 0.5 proposed by Fornell & Larcker [43]. Consequently, convergent validity was considered satisfactory, with all AVEs reaching 0.5 and beyond.

We established discriminant validity through Fornell and Larcker’s [43] approach, ensuring that the square root of the AVE for each construct exceeded the association between that construct and all others (see Table 3). Additionally, we evaluated discriminant validity using the Heterotrait-Monotrait (HTMT) ratio of association, a method confirmed to be more robust than Fornell and Larcker’s [43]. When HTMT values exceed 0.9, concerns about discriminant validity arise. However, all ratios remained below the indicated value of 0.9, confirming discriminant validity (see Table 3).

We established discriminant validity through Fornell and Larcker’s [43] approach, ensuring that the square root of the Average Variance Extracted (AVE) for each construct exceeded the correlations between that construct and all others (Table 3). Additionally, we evaluated discriminant validity using the Heterotrait-Monotrait (HTMT) ratio of correlations, a method considered more robust than Fornell and Larcker’s [43]. When HTMT values exceed 0.9, concerns about discriminant validity arise. However, as shown in Table 3, all ratios remain below the specified value of 0.9, confirming discriminant validity.

The bootstrapped R2 values indicated that PE, EE, SI and FC collectively accounted for 30% of the variance in BI. Moreover, PE, EE, SI, FC, and BI collectively contributed to 74% of the variance in use of ChatGPT.

Analyzing the bootstrapped path coefficients (refer to Table 4), it was observed that BI significantly and positively affected PE (β = 0.398, t = 6.346, *p* < 0.001), EE (β = 0.144, t = 2.596, *p* < 0.01) and SI (β = 0.445, t = 7.095, *p* < 0.001), thereby providing support for H1, H2, and H3. Surprisingly, FC exhibited a significant negative impact on BI (β = −0.204, t = 4.635, *p* < 0.001), leading to the rejection of H4. Moreover, the outcomes from the PLS-SEM analysis suggest a significant positive association between PE and the utilization of ChatGPT (β = 0.141, t = 4.489, *p* < 0.001), thus confirming H5. Additionally, the results indicated a modest significant positive effect of SI on ChatGPT usage (β = 0.070, t = 2.603, *p* < 0.01), providing support for H7. Nevertheless, effort expectancy (β = −0.042, t = 1.610, *p* = 0.107) and facilitating conditions (β = 0.001, t = 0.026, *p* = 0.979) did not demonstrate a direct and significant impact on ChatGPT usage, resulting in the rejection of H6 and H8. Finally, the findings underscored a robust positive and significant effect of BI on ChatGPT usage (β = 0.789, t = 27.366, *p* < 0.001), thereby supporting H9.

**Figure 2:**
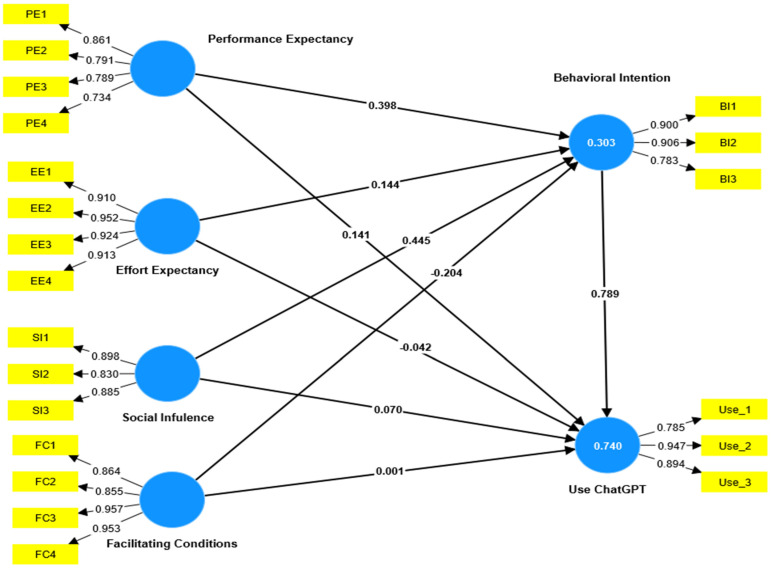
The examined research model.

Analyzing the bootstrapped (*n* = 5000) specific indirect effects showed that BIs mediate the link between PE and ChatGPT usage (β = 0.314, t = 6.076, *p* < 0.001), EE and ChatGPT usage (β = 0.114, t = 2.575, *p* < 0.05), SI and ChatGPT usage (β = 0.352, t = 6.708, *p* < 0.001), and between FC and ChatGPT usage (β = −0.161, t = 4.540, *p* < 0.001), supporting H10, H11,H12, and H13 (See Table 4 and Figure 2).

## 5. Discussion and Implications

This study aimed to understand higher education students’ acceptance and usage of ChatGPT for academic reasons, with a particular focus on students in public universities in SA. The results of a pre-tested survey drawing on the UTAUT framework showed interesting findings regarding the key determinants of ChatGPT usage for educational reasons. The results confirmed that the best determinant of ChatGPT use for academic reasons among SA students is SI. This is because students in SA are categorized as having a collective culture, which implies that the behavioral intentions of people are strongly shaped by their peers, family members, and colleagues [44]. The results showed that students have the intent to use ChatGPT because their colleagues are of the opinion that they should try this tool and recommend that they utilize ChatGPT for academic reasons. This finding is in line with the UTAUT’s assumption that SI has a significant impact on the BIs of technology acceptance [13]. These results are in agreement with previous studies [4,15,26,27] that revealed a positive significant influence of SI on ChatGPT use in higher education. This finding confirms that when peers and colleagues spread appropriate feedback about AI and its benefits for educational reasons, this positively affects other students’ BI to adopt ChatGPT for learning.

The findings revealed that the second-best predictor of BI to use ChatGPT for academic purposes among higher education students in SA is their PE. Students found ChatGPT a valuable tool for their academic pursuits as it allows them attain important and required information, enhances their productivity in academic studies, and elevates their academic performance. This outcome supports the results of Strzelecki [4], who also found that PE is the second-top determinant of students’ BI to use ChatGPT in education. This result is supported by other earlier studies, e.g., ref. [21], that also found a significant positive association between PE and BI to use ChatGPT in education. EE emerged as the third determinant of BI to use ChatGPT for academic purposes. This means that when students found ChatGPT easy to comprehend, easy to use, and easy to communicate with and it did not require much effort to use, they exhibited positive BI to use ChatGPT for academic reasons. This finding supports the work of Strzelecki [4] and Menon and Shilpa [15], who stated that when students perceive ChatGPT to be user-friendly, easily understandable, and seamlessly integrated into daily activities, they have a positive BI to use ChatGPT in their education. In addition, EE failed to have a direct significant influence on actual usage of ChatGPT in education. This means that when students perceive ChatGPT as user-friendly, easy to communicate with, and not requiring much effort, they are less likely to use ChatGPT directly, despite having positive BI to use it (which in turn affects their actual usage of ChatGPT for learning).

Unlike the UTAUT assumption [13] and previous research findings, e.g., ref. [4], which showed no significant influence of FC on BI to use ChatGPT in education, the current study showed negative significant influence of FC on BI to use ChatGPT in education among SA students. This is because students in universities in SA were not equipped with the necessary resources to make use of ChatGPT, they did not find themselves proficient in utilizing ChatGPT, and they did not receive support from their university in relation to it. However, when students had proper support from their institutions (as related to ChatGPT use in their educations) they exhibited positive BI to use it. Similarly, FC was found to have no significant influence on the actual use of ChatGPT. This contradicts the UTAUT framework [13] and previous findings [4]. The current study confirms that when students did not receive proper support from their tutors and institution regarding ChatGPT, they exhibited negative BI to use ChatGPT and would not use ChatGPT in their education.

With regard to indirect paths, aligning with UTAUT model [13] and the results of Strzelecki [4], BI was found to mediate the link between PR, SI, EE, FC and actual ChatGPT usage in higher education for academic reasons. More specifically, BI was found to partially mediate the relationship between PE, SE, and ChatGPT usage in education. Nonetheless, it had a full mediating effect on the link between EE and the use of ChatGPT for academic reasons. Furthermore, BI had a significant negative mediating effect on the relationship between FC and actual use of ChatGPT in education. Again, lack of available resources and support given to students made them develop negative BI and had an insignificant effect on their ChatGPT usage in education.

The findings of this study have several theoretical and practical implications. Theoretically, the study confirmed that SI is best predictor of students’ acceptance and use of ChatGPT for academic purposes. This is mainly due to the cultural impacts on students in SA, whose behavior is shaped by their families and colleagues. Notwithstanding this, their colleagues showed positive behavior towards the use of ChatGPT for academic purposes. Other predictors included PE and EE on the BI of students to use ChatGPT for learning. Interestingly, FC was found to negatively affect students’ BI to use ChatGPT for academic purposes and had no significant effect on students’ actual use of ChatGPT for academic purposes. These results support neither the UTAUT framework nor previous research on the use of ChatGPT for academic purposes [4]. However, these results call for higher education institutions to better equip their students with the required resources and support if they would like to integrate ChatGPT as a tool for supporting learning in higher education. Higher education institutions should develop a mechanism that encourages the responsible use of AI tools, including ChatGPT, for academic purposes among their students. Hence, necessary resources and support, such as a specialized support unit, should be made available to students. It is also important that leaders of higher education institutions launch awareness campaigns to raise the awareness of their students about the shortcomings of AI tools in general, including ChatGPT. This should include both the positive and negative impacts of using AI tools for academic purposes. They need to raise the accountability of their students in relation to the responsible and ethical use of these tools [45].

## 6. Limitations and Future Research Directions

This study was undertaken on a sample of management students in a public higher education institution in SA. The results showed some interesting findings, which supported and contradicted the UTAUT framework. However, the sample of study did not include participants from different institutions (including private sector institutions), who may have different perceptions of integrating AI tools like ChatGPT into education. Therefore, future research could address this research opportunity. This research did not study the moderating influence of participants’ characteristics such as gender, age, specialization, study year, and experience on the adoption of technology in education. This could be another venue for investigation in future studies. A comparison between undergraduate and postgraduate students could also be undertaken in future studies in different countries.

## 7. Conclusions

This study responds to the need to examine students’ acceptance and usage of ChatGPT for academic reasons due to the growing interest in this technology among students since its inauguration in 2022. This study advances our understanding on the variables that affect students’ acceptance and usage of ChatGPT for academic purposes. The results partially support the UTAUT framework and previous research in relation to the significant direct impact of PE, SI and EE on BI to use ChatGPT and the significant direct effect of PE, SI and BI on actual use of ChatGPT. Nonetheless, the results did not support previous studies in relation to the direct effect of FC on both BI and actual use of ChatGPT, which was found to be negative in the first relationship and insignificant in the second relationship. Therefore, there was partial mediation of BI in the link between PE, SI, and FC and actual use of ChatGPT in education. However, there was full mediation in the link between BI and EE and actual use of ChatGPT in education. The results of study confirm a need to support and equip students with the necessary resources to make more effective use of ChatGPT for academic purposes. It is important that higher education institutions ensure that their students recognize the shortcomings of AI tools and their long-term impacts on students’ learning to maintain sustainable learning outcomes. Hence, they should encourage responsible and ethical use of ChatGPT for academic purposes.

## Figures and Tables

**Table 1:** Students’ profiles (N = 520).

Profile		Freq.	%
Gender	Male	285	54.8
	Female	235	45.2
Age	Less than 20 years	241	46.3
	20 to 25 years	267	51.3
	26 to 30 years	12	2.4
Study level	Freshman (year one)	116	22.2
	Sophomore (year two)	123	23.7
	Junior (year three)	147	28.3
	Senior (year four)	134	25.8
	No	113	21.7

**Table 2:** Dimensions and variables’ psychometric properties.

	Scale Variables and Items	Loadings	VIF
PE: (α = 0.806, CR = 0.815, AVE = 0.632)			
PE1	“ChatGPT is a valuable tool for my academic pursuits”	0.861	1.613
PE2	“Utilizing ChatGPT improves the probability of attaining important objectives in your academic pursuits”	0.791	1.710
PE3	“ChatGPT enhances productivity in academic studies by expediting the completion of tasks and projects”	0.789	1.523
PE4	“Using ChatGPT can elevate my academic performance”	0.734	1.613
EE: (α = 0.944, CR = 0.959, AVE = 0.855)			
EE1	“I find it easy to learn how to use ChatGPT”	0.910	4.033
EE2	“Communication with ChatGPT is transparent and easy to comprehend”	0.952	4.832
EE3	“ChatGPT is user-friendly and intuitive”	0.924	4.256
EE4	“I find it effortless to acquire expertise in using ChatGPT”	0.913	3.679
SI: (α = 0.850, CR = 0.904, AVE = 0.760)			
SI1	“People who play a crucial role in my life are of the opinion that I should utilize ChatGPT”	0.898	1.806
SI2	“People who shape my behavior recommend the utilization of ChatGPT”	0.830	2.287
SI3	“Those whose opinions I hold in high esteem suggest that I make use of ChatGPT”	0.885	2.835
FC: (α = 0.939, CR = 0.950, AVE = 0.825)			
FC1	“I am adequately equipped with the necessary resources to make use of ChatGPT”	0.864	3.984
FC2	“I am proficient in utilizing ChatGPT due to acquired knowledge”	0.855	4.035
FC3	“ChatGPT is suitable for the technologies I utilize”	0.957	4.157
FC4	“When facing difficulties with ChatGPT, it is possible to receive support and aid from external sources”	0.953	3.984
BI: (α = 0.831, CR = 0.855, AVE = 0.747)			
BI1	“I have decided to continue using ChatGPT in the times ahead”	0.900	2.366
BI2	“I am dedicated to utilizing ChatGPT as a tool for my studies”	0.906	2.343
BI3	“I aim to continue using ChatGPT on a frequent basis”	0.783	1.572
Actual Use (AU) (α = 0.848, CR = 0.853, AVE = 0.771)			
AU1	“I intend to use the knowledge and skills I acquired from the ChatGPT in my educational activities”	0.785	1.578
AU2	“The knowledge and skills I acquired from the ChatGPT will be useful to me in class”	0.947	4.134
AU3	“Using ChatGPT has helped to improve my academic performance”	0.894	4.109

**Table 3:** Discriminant validity based on the Fornell and Larcker and HTMT methods.

	BI	EE	FC	PE	SI	Usage
BI	**0.864**					
EE	0.058 [0.73]	**0.925**				
FC	−0.171 [0.154]	0.450 [0.523]	**0.908**			
PE	0.297 [0.354]	0.166 [0.188]	−0.027 [0.066]	**0.795**		
SI	0.319 [0.361]	−0.136 [0.159]	−0.046 [0.045]	−0.293 [0.354]	**0.872**	
Usage	0.801 [0.195]	0.017 [0.044]	−0.160 [0.141]	0.348 [0.417]	0.286 [0.312]	**0.878**

Bold figures show the square root of AVE,; HTMT ratios are shown in brackets.

**Table 4:** Path coefficient and related *t* and *p* values.

Paths	Path Coefficient	*t* Statistics	*p* Values	Results
PE -> BI [H1].	0.398	6.346	0.000	Accepted
EE -> BI [H2].	0.144	2.596	0.009	Accepted
SI -> BI [H3].	0.445	7.095	0.000	Accepted
FC -> BI [H4].	−0.204	4.635	0.000	Rejected
PE -> ChatGPT usage [H5].	0.141	4.489	0.000	Accepted
EE -> ChatGPT usage [H6].	−0.042	1.610	0.107	Rejected
SI -> ChatGPT usage [H7].	0.070	2.603	0.009	Accepted
FC -> ChatGPT usage [H8].	0.001	0.026	0.979	Rejected
BI -> ChatGPT usage [H9].	0.789	27.366	0.000	Accepted
Specific indirect paths				
PE -> BI -> ChatGPT usage [H10].	0.314	6.076	0.000	Accepted
EE -> BI -> ChatGPT usage [H11].	0.114	2.575	0.010	Accepted
SI -> BI -> ChatGPT usage [H12].	0.352	6.708	0.000	Accepted
FC -> BI -> ChatGPT usage [H13].	−0.161	4.540	0.000	Accepted

## Data Availability

Data are available upon request from researchers who meet the eligibility criteria. Kindly contact the first author privately through e-mail.
